# A Dead-End Host: Is There a Way Out? A Position Piece on the Ebola Virus Outbreak by the International Union of Immunology Societies

**DOI:** 10.3389/fimmu.2014.00562

**Published:** 2014-10-27

**Authors:** Clive M. Gray, Marylyn Addo, Reinhold E. Schmidt, for the Clinical Immunology Committee of the IUIS

**Affiliations:** ^1^Division of Immunology, Institute of Infectious Diseases and Molecular Medicine, National Health Laboratory Services, University of Cape Town, Cape Town, South Africa; ^2^Division of Emerging Infections/Tropical Medicine, Department of Medicine, University Medical Center Hamburg-Eppendorf, Hamburg, Germany; ^3^Division of Immunology and Rheumatology, University of Hannover, Hannover, Germany

**Keywords:** vaccines, Ebolavirus, immunotherapies, clinical trials, immunopathogenesis

The fact that not everyone with Ebola virus disease (EVD) has died during the ongoing outbreak in West Africa, with an estimated case fatality rate of 70.8% by September 2014 (1), suggests that some kind of immunity to this virus is possible. If left unchecked, this scenario will undoubtedly shift to a higher figure, as health-care conditions in many of the countries affected may not always enable infected hosts to recover. Although gender differences in the survival, incidence, and/or severity of infection are unknown, the current Ebola virus outbreak represents an unprecedented disaster for humans (1, 2) and a zoonotic successful strategy (3) for a virus that cunningly and rapidly hijacks innate immunity to devastating effect. Human to human transmission is via contact with bodily fluids from symptomatic patients, which was identified in the first epidemic almost 40 years ago, but unlike prior epidemics, which consisted of sporadic short periods of human transmission, there is now a shift to a prolonged period of viral transmission (over 9 months at the time of writing). This is, in part, a reflection of the movement of people into urban densely populated regions (2). In fact, there are distinct parallels between the first outbreak in the Zaire with today’s outbreak – both being caused by Zaire Ebolavirus (1, 4). Importantly, however, the current and ongoing outbreak is occurring in this region for the first time, and there are fears that without blocking transmission, Ebola may become endemic (2). What then are the medical efforts under way to halt the spread of Ebola with immune therapies and vaccine strategies? Should we temper the usual scientific rigor of clinical trials and roll-out candidate vaccines and therapies directly into people? How should the scientific, and specifically the immunology community, meet the rapidity of EVD spread, which without doubt is the major challenge and crisis of today.

Ebola, a filovirus, encodes seven genes: nucleoprotein (NP), VP35 (polymerase co-factor), VP40 (matrix protein), glycoprotein (GP), VP30 (transcription activator), VP24 (secondary matrix protein), and RNA-dependent RNA polymerase (5). The most likely routes of Ebola entry into the body are via mucosal surfaces, the conjunctiva, the oropharynx, or injured skin routes (6). From human and monkey studies, the primary targets are dendritic cells, monocytes, macrophages, and Kupffer cells in the liver and entry mechanisms include the GP interacting with an unidentified receptor and inducing: (i) endocytosis mediated by lipid rafts; (ii) macropinocytosis; and (iii) clathrin-mediated transport (5). Experimental models have shown that virus-like particles (VLPs) composed of GP and VP40 activate endothelial cells that upregulate expression of intercellular adhesion molecule 1 (ICAM-1), vascular cell adhesion molecule 1 (VCAM-1), and E-selectin, which jointly results in an increase in endothelial permeability (7). It is also likely that the increase in pro-inflammatory cytokines, shown experimentally and in plasma samples from patients with Ebola infection (5), contribute to enhancing vasodilation and the resulting cytokine storm (8) causes immune dysfunction and general “shut-down” of all immunity. Additionally, monkey models have shown that Ebola infection causes apoptosis in by-stander CD4 and CD8 lymphocytes and NK cells (9). These devastating effects result in the clinical symptoms of hemorrhagic lesions in the skin, mucous membranes, visceral organs, and large effusions in body cavities. There is also multifocal necrosis most predominantly in the liver, spleen, kidneys, testes, and ovaries (5).

As with all pathogens, Ebola has evolved to evade innate immunity by hijacking specific anti-viral pathways, resulting in immunosuppression. VP35 and VP24 inhibit type I interferon activity. VP35 inhibits induction of IFN-beta production by suppressing phosphorylation and dimerization of IFN regulatory factor 3 (IRF-3) and enhancing SUMOylation of IRF-7 (5, 10). Viral P24 inhibits type I and type II IFN signaling by inhibiting nuclear signaling transducer ad activator of transcription 1 (STAT 1) (10).

The combined effect of these immune evasion strategies along with eliciting acute immune activation, inflammation, and a cytokine storm is lethal for the host and, as this current epidemic has shown, more than two-thirds of infected people with EVD will die. Are there clues from individuals who have survived EVD or properties of the virus that can be exploited to develop vaccine candidates?

## What of Immunotherapy?

In spite of recurrent Ebola virus outbreaks over 38 years, immunotherapy, in the form of passive immunization, was never really a focus of either the research community or of big pharma. However, about a decade ago, the US army as well as the public health agency of Canada realized the importance of developing antibodies against this devastating virus and therefore funded a few research projects leading to monoclonal antibodies.

Firstly, if we examine ZMapp, comprising a combination of available monoclonal antibodies, i.e., c13C6 from MB-003 and two humanized monoclonal antibodies from ZMab, c2G4, and C4G7. This product is on the market, but now in very short supply or not at all. The antibody combinations have proven to provide effective protection against Ebola virus in non human primates (11, 12). MB-003, by itself, is a cocktail of three human or human–mouse chimeric monoclonal antibodies c13C6, h13F6, and C6D8. This cocktail was reported in September 2012 and tested in Ebola-infected rhesus macaques (11, 12). When these antibodies were provided to experimental animals 24 or 48 h after infection, four of six macaques survived with little viremia and only a few clinical symptoms. ZMab is a cocktail of three monoclonal mouse antibodies: M1H3, m2G4, and m4G7. They are directed against the Ebola virus surface glycoprotein (EBOV-GP). They were tested in four Ebola virus infected macaque monkeys 48 h after infection. Two of the animals survived. The antibodies contained in ZMapp are nowadays produced in the tobacco plant *Nicotiana benthamiana*, i.e., in a bioproduction process known as “pharming.” In a process called “rapid antibody manufacturing platform” (RAMP), tobacco plants are infected with viral vectors using Agrobacterium cultures. Later, the antibodies are extracted and purified from these plants. The company producing these antibodies is named Kentucky BioProcessing, a subsidiary of Reynolds American (11, 13).

Until the occurrence of the current Ebola outbreak, ZMapp had not been used in humans. However, in keeping with the Animal Efficacy Rule, the FDA provided permission to urgently treat sick people under the organization’s expanded access program. The accepted procedure for testing safety and efficacy of drugs has so far not been possible for this dangerous pathogen. ZMapp was made available for two health-care workers who were infected by Ebola during their work in Liberia. Of the few doses available, the two health-care workers from the US were infused with ZMapp serum and both survived. Another Spanish patient died of Ebola 2 days after receiving the drug. Three more patients were treated in Liberia of which one died. Lastly, a British nurse with Ebola underwent treatment in Sierra Leone and survived the infection (14, 15).

In summary, based on these few sporadic cases, their heterogeneity in outcome and adjunct therapies, the efficacy of this drug in patients cannot be accurately judged. In addition, ZMapp announced that the supply of this drug had been exhausted and so further assessment of the efficacy of this agent was no longer possible.

A second approach is the use of siRNA. The FDA has allowed the RNA interference drug known as “TKM-Ebola” for application in patients infected with Ebola. There is no experience in the field with this approach, or at least none has been reported. A Phase 1 trial in the US has started, but was transiently placed on clinical hold to further investigate a cytokine-mediated inflammatory response in a recipient receiving high dose TKM-Ebola.

A strategic use of passive immunotherapy could be in post-exposure prophylaxis. However, as the outbreak continues there is currently no time to conduct traditional clinical efficacy and safety studies before use in human subjects. Regardless of this situation, passive immunotherapy using monoclonal antibodies warrants further study for use in tandem with other biomedical interventions.

Other experimental therapy approaches include Favipiravir (T705), an anti-viral compound currently licensed for influenza outbreaks and brincidofovir, an experimental drug developed for the treatment of cytomegalovirus infections.

## What of Vaccines?

This is the most important strategy, which will mitigate ongoing viral transmission (21). Although to date there are no licensed vaccines available to prevent EVD, significant progress has been made in recent years. The following vaccine platforms are currently under development and have shown promise in pre-clinical screening against filoviruses such as Ebola virus (EBOV) and Marburg virus (MARV): VLPs, Venezuelan equine encephalitis virus replicons (VEEV RP), replication competent recombinant human parainfluenza virus 3 (rHPIV3), replication incompetent adenovirus vectors, and recombinant vesicular stomatitis virus (rVSV) (16, 17). However, only the latter two vaccine platforms are advanced enough to move into clinical trials and will be briefly discussed herein.

Recombinant Chimpanzee Adenovirus Serotype 3 Vectored Ebola Vaccine (CAd3 Ebola vaccine) is manufactured by Okairos/GSK (acquired by GSK in 2013). Notably, doses that are sufficient for Phase I clinical trials have been donated by the Vaccine Research Center, NIAID.

The recombinant adenovirus vector vaccine platform has been widely used in vaccine development for a host of infectious diseases, including HIV, malaria, HCV, and TB. The CAd3 Ebola vaccine is a viral vectored vaccine that expresses the Ebola glycoprotein (GP) and is a non-replicating vector based on an adenovirus that primarily infects chimpanzees in the wild. The use of a chimpanzee adenovirus alleviates concerns about high levels of pre-existing immunity to human adenoviruses that may likely reduce the immune response to some human adenovirus-based vaccines. There are two ChAd3 Ebola vaccines, one that is bivalent (against Zaire and Sudan strains), and one that is monovalent (Zaire only). Only the monovalent Zaire vaccine is currently being manufactured on a larger scale. A promising study in non-human primates (NHP) given a lethal dose of EVD, found that CAd3 protected all 16 animals with a single treatment. There is in fact already clinical trial experience in humans using this vaccine platform, with the CAd3 constructs coding for proteins against HCV and RSV, where it has been tested in 290 people. Related CAd63 vectors were also tested in clinical trials against malaria in more than 1000 individuals with no serious safety concerns. Phase 1 trials to assess the safety and immunogenicity of this vaccine construct have started in the US and UK in September 2014 with a roll-out planned for African sites (non-EVD-endemic for Phase I) shortly. As such, implementation of these approaches in West Africa could be imminent.

Recombinant VSV-ΔG-ZEBOV was developed and sponsored by the Public Health Agency of Canada and BioProtection Systems Corporation (NewLink Genetics Corporation). The rVSV platform represents a promising vaccine candidate approach against filoviruses (18). VSV is a non-segmented, negative-stranded RNA virus in the family Rhabdoviridae, which is an animal pathogen and rarely causes serious disease in humans. Several VSV characteristics contribute to its favorable profile as a vaccine vector: replication in mammalian cell lines, growth to very high titers, and a strong induction of innate and adaptive (humoral as well as cellular) immune responses. This, coupled to the very low levels of pre-existing immunity to VSV in the general population, makes such a vector attractive. Furthermore, VSV induces a neutralizing antibody immune response primarily directed against the VSV glycoprotein (VSV-G), a characteristic, which was exploited for the design of the Ebola vaccine. The rVSVΔG-ZEBOV vaccine consists of a recombinant VSV, in which the G coat GP has been deleted and substituted with Ebolavirus Zaire envelope GP. The recombinant virus is highly attenuated and can be grown to high titers. The use of attenuated rVSV as a vaccine platform against filovirus has been demonstrated to be safe in mice and NHP. Animals vaccinated with, rVSV-ZEBOV-GP or rVSV-MARV-GP did not experience any major toxicity. In pre-exposure pre-clinical studies in 20 NHP, the vaccine showed 100% efficacy. Post-exposure prophylaxis led to survival of 50% of NHP. Although clinical data in humans is limited, the rVSVΔG-ZEBOV-GP vaccine has been administered to a German laboratory worker in the context of post-exposure prophylaxis after Ebola exposure by a needle stick injury and was well tolerated in this setting (19). Clinical trials of rVSV expressing HIV antigens are currently under way. The start of Phase 1 clinical trials to assess the safety and immunogenicity of rVSVΔG-ZEBOV-GP is imminent and are to be conducted in US, European, and African trial sites.

## Conclusions and the Way Forward

The fact that the current outbreak is through a single strain (Zaire-Ebola) and the mutability and variability of this virus are very low, immunotherapeutic cocktails and vaccine strategies should be easily achievable. Animal studies and sporadic clinical evidence suggest that these approaches may be successful. The question then becomes: how to speed up the process? Implementation without undergoing some form of clinical safety trials is perilous, due to the fact that not all people infected with Ebola virus die and placing humans at greater risk through a vaccine would not be an option. Conversely, those people who survive will have immunity and could be employed to implement vaccine cover.

This outbreak has demonstrated the fragility of public health systems in West African countries and breaking the human transmission chain by immunizing large numbers of people is ultimately going to halt viral spread and provide protection against future outbreaks. In line with WHO recommendations (20), the IUIS proposes that animal safety and immunogenicity trials should be performed in parallel, and as soon as possible, with human Phase I trials with small sets of volunteers to assess safety and to optimize dosage levels. Based on these data, the rapid roll-out of phase 2 trial designs is warranted, allowing for collection of further safety and immunogenicity data as well as early efficacy studies. As time is not on our side, there needs to be adequate financial support to bring vaccine candidates to clinical trials (21) and then a speedy implementation in African countries within a matter of weeks or months. Although not immediately critical to halting the current outbreak – an additional more long-term goal would be to identify the animal reservoir to prevent future zoonotic transmission.

## Conflict of Interest Statement

The authors declare that the research was conducted in the absence of any commercial or financial relationships that could be construed as a potential conflict of interest.
